# Does Smartphone Use Affect Attitudes Toward Aging Among Older Adults in Rural Areas? An Empirical Analysis Using Data from the Chinese Longitudinal Aging Social Survey

**DOI:** 10.3390/bs14111069

**Published:** 2024-11-08

**Authors:** Xiaohui Wang, Yifan Zhao

**Affiliations:** 1 School of Public Administration, Yanshan University, Qinhuangdao 066004, China; 2 Institute of Social Development, Chinese Academy of Macroeconomic Research, Beijing 100038, China; zhaoyifan19921022@163.com

**Keywords:** smartphone use, rural older adults, attitudes toward aging, active aging

## Abstract

Fostering positive attitudes toward aging among older adults serves as a key measure of success in promoting active aging. Based on microdata from the China Longitudinal Aging Social Survey in 2018, this study uses ordinary least squares (OLS) and the two-stage least squares (2SLS) to evaluate the impact of smartphone use on the attitudes toward aging among older adults in rural areas. The results show that smartphone use significantly relieves older adults’ negative attitudes toward aging, with effects persisting even after employing robust estimations and instrumental variable techniques to address endogeneity concerns. These results emphasize the need to improve rural internet infrastructure and to promote both smartphone access and literacy among older adults in rural China to amplify these positive effects.

## 1. Introduction

Population aging is a significant matter in today’s society, and it requires attention. As many young and middle-aged skillful individuals migrate from rural to urban areas, the aging population in rural areas is increasing. According to China’s seventh national census data, the population aged 60 and above counts for 264.02 million individuals, accounting for 18.70% of the total population. Additionally, the population aged 65 and older stands at 190.64 million, accounting for 13.5% of the total. In rural areas, the proportion of individuals aged 60 and older is 23.81%, while those aged 65 and older make up 17.72%. These figures are 7.99 and 6.61 percentage points higher, respectively, compared to urban areas [1]. The phenomenon of the “urban–rural inversion” of population aging will continue, and the discrepancy in aging between individuals who reside in urban and rural areas will increase. The large-scale older adults in rural areas have become a relatively vulnerable group due to their special physical functions and socioeconomic status. They often face more physiological and psychological challenges, with negative attitudes toward aging being a significant risk factor.

In general, attitudes toward aging are complex social psychological structures. They encompass individual perceptions, experiences, and evaluations of the aging process and the lives of older adults. Typically, these attitudes include three components: psychological and social loss, physiological changes, and psychological gain [2]. In relevant related studies, attitudes toward aging are often used interchangeably with age identification, subjective age, aging perception, and so on [3]. Attitudes toward aging can be categorized as either positive or negative based on their content. A positive attitude toward aging refers to a positive feeling toward old age, such as growth, wisdom, and more freedom brought about by age, reflecting an individual’s successful adaptation to the aging process. A negative attitude toward aging refers to the negative feelings that stem from aging. These can include declining health levels and social isolation. As an individual psychological factor of older adults, their attitudes toward aging can affect their cognitive abilities, behavioral patterns, and physical and mental health but also affect their quality of life. A positive attitude toward aging can help older adults alleviate their reactions to daily stressors [4], promote mental health and positive self-identity, have a longer average life expectancy [5], and have better well-being [6]. Older adults with a positive attitude toward aging are often able to maintain healthy behavioral habits, have stronger language expression and memory abilities [7], and are more receptive to changes in their social relationships. This attitude makes them more proactive in social activities and interactions [8]. However, negative attitudes toward aging are detrimental to the physical and mental health of older adults [9].

Older adults in traditional rural societies have a more positive attitude toward aging, possibly influenced by the traditional culture of showing respect to older adults [10]. As modernization progresses, older adults’ attitudes toward aging have also changed. Older adults’ attitudes toward aging are influenced by multiple factors. In terms of individual characteristics, age is negatively correlated with aging. Male-identifying older adults have a more positive attitude toward aging than those who are female-identifying. Older adults with higher levels of education are more able to actively cope with the issues of aging. In particular, suffering from chronic diseases can have a significant negative impact on their attitudes toward aging [11]. In terms of family characteristics, older adults with better economic conditions have a more positive attitude toward aging [12], while those in poverty tend to have negative attitudes toward aging [13]. What is more, the attitudes and opinions of family members toward older adults can influence their attitudes toward aging [14]. In addition to that, increasing emotional connections among family members can weaken the damage of negative aging attitudes toward older adults [15]. In this regard, children’s intergenerational support can have a positive effect on older adults’ attitudes toward aging [16]. Older adults who participate in the childcare of their grandchildren have a more positive attitude toward aging [17]. In terms of social characteristics, the attitudes of older adults toward aging are influenced by the quantity and quality of their social relationships [18]. Those with higher levels of social support tend to have a more positive attitude toward aging [19], while those who experience social isolation often have a more negative attitude [20].

Under the rapid development of the Internet and the widespread application of information technology, the number of older adults using the Internet has surged significantly. According to the 53rd Statistical Report on China’s Internet Development released by China Internet Network Information Center, the number of Internet users aged 60 and above in China reached 171 million by the end of 2023. That accounts for 15.6% of the total Internet users, and the Internet penetration rate of the population aged 60 and above will be 57.5% [21]. It is predicted that by the middle of the 21st century, the Internet will be widely used among the elderly, with a conservative estimate of 60% [22]. Among the various smart devices available, smartphones have become the primary means for older adults to access the Internet. According to the fifth sampling survey on the living conditions of older adults in urban and rural areas in China, 36.6% of older adults were using smartphones in 2021 [23]. More than 80% of older adults have become proficient in WeChat and have used it as a platform for social and emotional connections. Additionally, more than 50% frequently use their smartphones to pay their bills, and about 30% use them for navigation and commuting [24].

The existing literature has discussed older adults’ attitudes toward aging from different perspectives. However, research specifically addressing the impact of smartphone use on the attitudes of rural older adults toward aging remains limited. The influence of smartphones on the lives of older adults in rural areas is complex. Smartphones not only improve intergenerational communication between older adults and their children but also broaden their social engagement and entertainment options. The growing popularity of short videos and other media formats in rural areas has encouraged more older adults to connect with online communities and digital migrants through platforms such as “TikTok”, “Kwai”, and “ WeChat Moments”. These platforms not only fulfill their needs for entertainment, leisure, and learning but also expand their social networks, ultimately enhancing their quality of life [25,26]. The widespread popularity of employment platforms represented by e-commerce in rural areas has also encouraged some older adults in rural areas to enter the labor market and exert their residual energy [27]. In addition, in these areas where information is relatively limited and medical resources are relatively scarce, the use of smartphones enables older individuals to access health resources and medical services more conveniently. All of the above may affect their attitudes toward aging. Based on these, this study takes rural older adults as the research subject and analyzes the impact of smartphone use on attitudes toward aging based on an empirical approach. Additionally, it proposes the following hypothesis: Smartphone use can relieve older adults’ negative attitudes toward aging. This research holds practical significance for promoting the adoption of information technology, implementing active aging strategies, and enhancing the subjective well-being of rural older adults. Additionally, it aims to contribute micro-empirical evidence to the existing body of research in this field.

## 2. Materials and Methods

### 2.1. Data

This study used data from the China Longitudinal Aging Social Survey (CLASS), which was designed by the Institute of Geriatric at Renmin University of China and implemented by the China Survey and Data Center of Renmin University of China. It employed a hierarchical multi-stage probability sampling method to gather information on various factors related to older adults aged 60 and above, including their socio-economic status, health and related services, care planning, social support, and both physical and mental well-being. Specifically, county-level regions were selected as primary sampling units (PSUs), and village communities or neighborhood committees were selected as secondary sampling units (SSUs). During the investigation, the project team used the map method to draw and number all buildings in the city and the villages, listing all residential and household information and extracting samples. The survey covered 28 provinces (including cities and autonomous regions) in China, except Hong Kong, Macao, Taiwan, Hainan, Xinjiang, and Xizang. It is a representative sample and currently recognized as authoritative data with scientific research value by the academic community. This study selected the 2018 class data for analysis, as they are the most recently released data and reflect the conditions following the implementation of the rural revitalization strategy. The survey sample for that year included 11,418 individuals from 462 villages and neighborhoods across 134 counties nationwide. This study focused on older adults aged 60 and above living in rural areas. After data cleaning, 4816 valid samples were retained.

### 2.2. Variables

#### 2.2.1. Dependent Variable

The dependent variable of this study was older adults’ attitudes toward aging. These are relatively complex social psychological structures, and there is still no unified standard for measuring them among domestic and foreign scholars. The commonly used scales include the Attitudes Toward Aging Questionnaire (AAQ), Attitude Toward Own Aging (ATOA), Single-Category Implicit Association Test (SC-IAT), Facts on Aging Quiz (FAQ), and Kogan’s Attitudes toward Older People (KAOP). There are 12 questions related to the dependent variable from CLASS in 2018, but these questions cannot be fully used as a scale of any type. There is also strong subjectivity and the accumulation of measurement errors in the synthesis of multiple variables, which leads to serious endogeneity problems. Psychological and social loss is the most important indicator in various scales, reflecting the negative psychological and social functional experiences perceived by older adults in the aging process. Given this, and combined with the availability of data, this study uses the core question in the questionnaire of “Did you feel useless in the past week?” as a measure of attitudes toward aging. The answers are “no”, “sometimes”, and “often”, with values assigned as 1, 2, and 3 in sequence. A higher value indicates a more negative attitude toward aging. The practice of using a single-dimensional variable to characterize the subjective feelings of older adults is relatively common in the existing literature, which can succinctly convey the psychological state of older adults and avoid endogeneity issues caused by the synthesis of too many subjective indicators as much as possible [28,29].

#### 2.2.2. Independent Variable

The core explanatory variable of this study was smartphone use. Therefore, the chosen question in the questionnaire was “Do you currently use a smartphone?”, and a value of 1 was assigned to the answer “Yes”, and 0 to the answer “No”.

#### 2.2.3. Control Variables

Referring to the existing research, individual characteristic variables and family and social characteristic variables that affect older adults’ attitudes toward aging will be included in the regression model. In addition, dummy variables in the regions where older adults are located were included in the model to control for regional differences (variable selections and descriptions can be found in Table 1). A descriptive analysis of the variables is shown in Table 1.

In Table 1, it can be seen that the answers of rural older adults to the core question “Did they feel useless in the past week?” were 43.36%, 42.24%, and 14.40% for “never”, “occasionally”, and “often”, respectively. The average score of aging attitudes was 1.710, indicating that the majority of older adults expressed good attitudes toward aging. The usage rate of smartphones among older adults in rural areas was very low, with only 10.71% of them using smartphones. In Table 2, it can be seen that the average score of attitudes toward aging for rural older adults who use smartphones was 1.519. Additionally, the average score of attitudes toward aging for those who do not use smartphones was 1.734. This preliminary indicates that rural older adults’ attitudes toward aging who use smartphones are better than those who do not use smartphones.

In the overall sample, older adults identifying as male made up 53.28%, while those identifying as female accounted for 46.72%. The overall educational attainment of older adults was relatively low, with 38.70% lacking formal education. Additionally, 44.33% had completed primary school, and 13.68% had reached the middle school level, while only 3.28% had graduated from high school or attained a higher level of education. Nearly three-quarters of the older adults suffered from one or more chronic diseases, representing 73.82% of the population. A total of 67.40% were married. On average, each older adult had 3 surviving children, and 2.65 individuals lived in the same household. Furthermore, 60.26% of the older adults were responsible for caring for their grandchildren, and 88.68% benefitted from pension security.

### 2.3. Measurement Model

Given that the dependent variable—older adults’ attitudes toward aging—is a ranking variable, the existing research indicates that when models are appropriately specified, there is no inherent superiority or inferiority between OLS and ordered logit models [30]. Therefore, this study uses the commonly used OLS to examine the impact of smartphone use on older adults’ attitudes toward aging. The model is constructed as follows:

(1) $${Aging}_{i}=\alpha_{0}+\beta_{1}{Smartphone}_{i}+\gamma X_{i}+{{prov}_{i}+\varepsilon }_{i}$$

In Equation (1), ${Aging}_{i}$ represents the older adults’ attitudes toward aging, ${Smartphone}_{i}$ indicates whether older adult i uses smartphones, $X_{i}$ represents other control variables, $\alpha_{0}$, $\beta_{1},$ and γ is the coefficient to be estimated, where γ is in vector form, ${prov}_{i}$ is a dummy variable for the province, and $\varepsilon_{i}$ is the error term.

## 3. Results

### 3.1. OLS Results of Smartphone Use on Rural Older Adults’ Attitudes Toward Aging

Table 3 presents the regression results examining the impact of smartphone use on the attitudes toward aging among older adults in rural areas. Model (1) included only smartphone use indicators to examine the impacts of key explanatory variables on their attitudes. Models (2) and (3) progressively incorporated individual characteristic variables, as well as family and social characteristic variables.

The results of Model (1) indicate that smartphone use had a significant negative impact on the attitudes of older adults in rural areas toward aging. However, after adding individual, family, and social characteristic variables in Models (2) and (3), the marginal effect of smartphone use on attitudes was diminished. Despite this decrease, the effect remained significantly negative at the 1% level. Specifically, compared to older adults who do not use smartphones, those who do experienced a 16.6% reduction in social and psychological difficulties, indicating that rural older adults who use smartphones tend to have a more positive attitudes toward aging. Therefore, the hypothesis is verified.

Among other control variables, age and the prevalence of chronic diseases significantly influenced rural older adults’ attitudes toward aging at the 1% statistical level. Specifically, for each additional year of age, the attitudes of rural older adults toward aging increased by 0.5%. Additionally, older adults with chronic diseases show a 15.6% improvement in their attitudes compared to those without chronic conditions. Regarding family and social characteristics, married older adults and those living with a larger number of permanent residents tended to exhibit lower positive attitudes toward aging. Conversely, rural older adults who received pension security demonstrated a 7.8% increase in their positive attitudes compared to those who did not have such support.

### 3.2. IV Regression Results of Smartphone Use on the Focus Sample

Considering the potential for reverse causality between smartphone use and rural older adults’ attitudes toward aging—specifically, that older adults with more positive attitudes toward aging are more likely to engage in social activities [31], i.e., use smartphones—it is important to clarify the relationship. To ensure the validity of the conclusions and the reliability of the data, we used the presence of network signals (wired or wireless) in the homes of older adults as the instrumental variable in the questionnaire. This variable meets the criteria of correlation and homogeneity: whether a residential house has network signals reflects the construction of internet infrastructure in a region, which can directly affect the smartphone use of older adults without directly affecting their attitudes toward aging. Table 4 shows the regression results of instrumental variables. When using the traditional two-stage least squares (2SLS) method, the regression coefficient of smartphones is significantly negative, and the model rejects the null hypothesis of unrecognizable and weak instrumental variables. When further adopting the robust maximum likelihood estimation method (LIML) with weak instrumental variables and the more effective GMM method under heteroscedasticity conditions, the regression results are consistent with the 2SLS method. It should be noted that when using the instrumental variable method, the marginal effect of smartphone use on older adults’ attitudes toward aging is greater than the estimated results of the OLS model. At the same time, the DWH test was passed, indicating potential endogeneity issues in the model. However, when using the instrumental variable method for estimation, the results are still robust.

### 3.3. Further Testing of the Results Using Another Variable or Method

At this stage, the core independent variable was replaced in sequence with older adults’ use of the internet. The dependent variable was also replaced with older adults who feel that they are still useful to society and the ordered logit model was used for estimation.

(1) Replacing the independent variable: According to the 49th Statistical Report on the Development of the Internet in China, 99.5% of older netizens use mobile phones to access the Internet. In this way, this function of smartphones has become the most popular amongst this group of people. Whether or not they use the Internet can, to some extent, reflect the usage of smartphones by older adults. Therefore, this study selected the indicator of older adults’ internet usage as a substitute for smartphone use for robustness testing. In the survey questionnaire, we assigned a value of 0 to the response “I never go online” and a value of 1 to the response “How many times a year do you go online?”, which included options such as “at least once a month”, “at least once a week”, and “every day”. The regression results are presented in Model (1) in Table 5.

(2) Replacing the dependent variable: The previous text portrayed the attitudes toward aging from the negative perspective of social psychological loss. Attitudes toward aging can also be assessed from a positive perspective, reflecting older adults’ positive perceptions of their self-worth. We selected the question “I think I am still a useful person to society” from the questionnaire and assigned the answers “completely disagree”, “somewhat disagree”, “average”, “somewhat agree”, and “completely agree” values of 1–5. respectively. The regression results are shown in Model (2) in Table 5.

(3) Updating the research methods: Since the dependent variable of “attitude to aging” was an ordered categorical variable with a value of 1–3, an ordered probability model (ordered logit model) was used for re-estimation. Table 5 shows the regression results using the ordered logit method for Model (3).

Based on the estimation results of Models (1)–(3), the research conclusions of this study can be considered robust.

## 4. Discussion

As a significant medium of the Internet, smartphones are increasingly influential among older adults. The existing literature provides limited insight into the relationship between smartphone use and older adults’ attitudes toward aging. Utilizing microdata from the China Longitudinal Aging Social Survey in 2018, this study empirically analyzed the impact of smartphone use on the attitudes toward aging among older adults in rural areas. Studies on the subject have found that smartphone use can significantly relieve older adults’ negative attitudes toward aging. In comparison to older adults who do not use smartphones, older adults in rural areas who use smartphones experienced a 16.6% decrease in social and psychological difficulties. This indicates that the probability of older adults in rural areas who use smartphones having a more positive attitude toward aging was increased by 16%. After addressing potential endogeneity issues through the instrumental variable method and passing a series of robustness tests, this conclusion remains valid. This is consistent with previous research on the association of Internet use with the social adaptation of older adults [32,33,34]. However, this study further focused on smartphone use among older adults in rural areas, using the indicator of psychological and social loss to more accurately reflect attitudes toward aging. How does smartphone use influence the attitudes of older adults in rural areas toward aging? The existing literature offers several explanatory pathways.

Firstly, as a social communication medium, the real-time interactive function of smartphones greatly reduces communication costs, undoubtedly creating a more convenient and efficient social channel for older adults. In this way, familial and geographical relationships that may have been diluted due to individualization and atomization are maintained [35]. The quality of life of older adults is significantly correlated with the scale of the social networks they develop. Additionally, individuals with a high degree of closeness, low heterogeneity, and strong relationships in the “core network” can receive sufficient spiritual support and abundant material resources. The lack of social networks or channels for integration into society can easily lead to a sense of social isolation [36]. As their age increases, the mobility of older adults gradually decreases, and older adults face many difficulties in participating in traditional social activities that require their presence. In addition, an increasing number of rural populations are flowing to cities. Additionally, rural communities are constantly dissolving. This has led to the disintegration of older adults’ original social networks, resulting in a continuous reduction in network size. Smartphones, equipped with features for interpersonal connections and intergenerational communication, have strengthened and broadened the social networks of older adults in rural areas [37], improving their attitudes toward aging.

Furthermore, as a channel for information acquisition, smartphones can expand and improve individual information acquisition capabilities. Through smartphones, older adults can actively obtain health information and learn about disease prevention methods based on their needs and preferences [38]. A survey from the Pew Internet and American Life Project showed that 80% of health seekers can find the information they need on the Internet [39]. For rural areas with relatively limited access to information and underdeveloped medical resources, smartphone use enables older adults to access health information and medical services more easily. Smartphone use is likely to affect their attitudes toward aging by improving their health status.

Moreover, as a technology that is used daily, smartphones provide more ways of learning, entertainment, leisure, and consumption. Therefore, smartphone use has increased the social participation of people and promoted their social integration [40]. Online social and entertainment activities can greatly alleviate the loneliness of older adults and enhance their sense of happiness [41,42]. For example, watching short videos, chatting online, making video calls, and voting online have become important activities for older adults through platforms, and their daily activities have become more colorful [43].

Based on the findings of this study, promoting smartphone use among older adults in rural areas is an important strategy for improving their attitudes toward aging. However, due to challenges related to their physiological conditions, education levels, and socio-economic status, many rural older adults have yet to experience the convenience and efficiency that smartphones offer. Numerous studies, both domestically and internationally, have confirmed a decline in the usage rate of smart products with age [44]. Even those rural older adults who have overcome the “access gap” still face various challenges while using smartphones and often lack confidence in their ability to learn and use them, leading to anxiety. Beyond the individuals themselves, the complexity of smartphone design also poses a significant barrier. To enhance the proficiency of rural older adults in smartphone use, it is essential to develop simpler and more user-friendly apps and provide digital technology training tailored for this demographic. Notably, as more Internet natives age, the smartphone use patterns of older adults in rural areas will increasingly resemble those of younger generations, significantly improving their attitudes toward aging.

There are some limitations included in this paper. Older adults in rural areas exhibit distinct characteristics in their smartphone use, including usage time, functions, and effects. The impact of different types of smartphone use on attitudes toward aging among this demographic may also differ. However, due to data limitations, this study could not differentiate between types of smartphone use among older adults in these areas. Future research should segment older adults based on their smartphone use patterns. Additionally, while this cross-sectional study offers valuable insights into mediating processes, longitudinal data may better capture shifts in attitudes toward aging over time and address potential selection bias in smartphone use (e.g., older adults who are more socially active or in better health may be more inclined to adopt smartphone technology). Future studies should utilize longitudinal data to comprehensively analyze smartphone use and its impact on attitudes toward aging among older adults in rural areas.

## 5. Conclusions

This study demonstrates that smartphone use significantly enhances the attitudes of older adults in rural areas toward aging. However, the smartphone adoption rate among this demographic remains low, with only 10.71% using these devices. Given the widespread digital divide affecting older adults across various countries, governments should prioritize increasing both the willingness and ability of older adults in rural areas to engage with smartphones. This strategy will further amplify the positive effects of smartphones on their attitudes toward aging.

On one hand, it is essential to accelerate the development of internet infrastructure in rural areas, as this serves as the foundation for older adults to utilize smartphones. However, a significant digital divide between urban and rural areas persists in the current internet infrastructure. Governments at all levels should expedite the expansion of broadband and mobile communication networks in rural regions, substantially enhance internet service capacity and performance, reduce household broadband and mobile communication fees, and promote the widespread adoption of smartphones among older adults in these areas. Additionally, efforts should focus on enhancing the willingness and ability of older adults in rural areas to use smartphones. Establishing a national training center for technology operation will provide older adults with guidance and support aimed at improving their information technology skills and the operation of smart devices. Recognizing the diversity of how smartphone use impacts attitudes toward aging is crucial, with particular emphasis on training programs for younger and less educated individuals. What is more, emphasizing “digital feedback” is also important. Children and younger generations should assist older adults by helping them view their situation positively and patiently explaining the operation of smart devices. Last but not least, exploring and promoting a “partner learning” approach can further enhance this effort. Guiding older adults to pair based on their social networks can help form mutual learning groups, encouraging those who have already mastered internet skills to share their experiences and foster a supportive digital environment.

## Figures and Tables

**Table 1:** Descriptions of study variables.

Category	Variable	Measurement	Sample Size	Mean	Standard Deviation
Dependent Variable	Attitudes toward aging	Never = 1	1945	1.710	0.703
		Occasionally = 2	1895		
		Often = 3	646		
Independent Variable	Smartphone use	No = 0	4300	0.107	0.309
		Yes = 1	516		
Characteristic Variables	Gender	Female = 0	2250	0.533	0.499
		Male = 1	2566		
	Age	Age	4816	71.570	7.252
	Education	Uneducated = 0	1864	4.295	3.687
		Primary School = 6	2135		
		Junior High School = 9	659		
		High School = 12	144		
		Junior College = 15	8		
		Bachelor’s Degree and Above = 16	6		
	Household Registration	Non-Agricultural Residence = 0	259	0.946	0.226
		Agricultural Residence = 1	4557		
	Chronic Diseases	No = 0	1261	0.738	0.440
		Yes = 1	3555		
Family Characteristic Variables	Marriage	Without a Spouse = 0	1570	0.674	0.468
		Having a Spouse = 1	3246		
	Family Size	Number of Family Members Who Often Live Together	4810	2.646	1.261
	Number of Children	Number of Surviving Children	4670	3.000	1.387
	Care For Grandchildren	No = 0	1914	0.603	0.489
		Yes = 1	2902		
Social Characteristic Variables	Old-Age Security	Yes = 0	545	0.887	0.317
		No = 1	4271		

**Table 2:** Smartphone use and their attitudes toward aging among older adults.

Panel A: Distribution of smartphone use and attitudes toward aging among older adults.			
	Never	Occasionally	Often
Use of smartphone	55.01%	38.04%	6.95%
No use of smartphone	41.93%	42.76%	15.31%
Panel B: Distribution of smartphone use and attitudes toward aging among older adults.			
	Sample size		Attitudes toward aging
Use of smartphone	489		1.519
No use of smartphone	3997		1.734
D-value	-		0.214 ***

Note: *** *p* < 0.01.

**Table 3:** Associations between smartphone use and attitudes toward aging.

Variable	Model (1)	Model (2)	Model (3)
Smartphone use	−0.211 *** (0.032)	−0.165 *** (0.033)	−0.166 *** (0.033)
Gender		−0.021 (0.021)	−0.013 (0.022)
Age		0.007 *** (0.002)	0.005 *** (0.002)
Education		−0.005 (0.003)	−0.003 (0.003)
Household Registration		−0.016 (0.049)	−0.013 (0.050)
Chronic Diseases		0.168 *** (0.025)	0.156 *** (0.026)
Marriage			−0.058 ** (0.025)
Family Size			−0.024 *** (0.008)
Number of Children			0.008 (0.009)
Care for Grandchildren			0.008 (0.021)
Old-Age Security			0.078 ** (0.034)
Regional Fixed Effect	Controlled	Controlled	Controlled
Constant	1.657 *** (0.046)	1.080 *** (0.137)	1.214 *** (0.152)
Sample Size	4486	4485	4351
F Statistics	61.35 ***	41.17 ***	39.86 ***
R^2^	0.0898	0.1065	0.1133

Note: The data in parentheses represent robust standard errors. The same applies below. ** *p* < 0.05 and *** *p* < 0.01.

**Table 4:** IV regression results.

Variable	Model (1) 2SLS		Model (2) LIML		Model (3) GMM	
	First Phase	Second Phase	First Phase	Second Phase	First Phase	Second Phase
Smartphone Use		−0.319 *** (0.094)		−0.319 *** (0.094)		−0.319 *** (0.094)
Network Signal	0.281 *** (0.015)		0.281 *** (0.015)		0.281 *** (0.015)	
Control Variables	Controlled	Controlled	Controlled	Controlled	Controlled	Controlled
Regional Fixed Effect	Controlled	Controlled	Controlled	Controlled	Controlled	Controlled
Statistics	F = 372.03 ***	F = 27.03 ***	F = 27.15 ***	Wald = 981.34 ***	F = 27.15 ***	Wald = 981.34 ***
Sample Size	4351		4351		4351	
DWH Test	2.907 *		-		-	

Note: * *p* < 0.1 and *** *p* < 0.01. The Kleibergen Paap rk LM statistic for model (1) was 310.707, rejecting the unrecognizable null hypothesis. The Kleibergen Paap rk Wald F statistic was 766.083, rejecting the null hypothesis of the weak instrumental variables.

**Table 5:** Robust check.

Variable	Model (1) Replace the Independent Variable	Model (2) Replace the Dependent Variable	Model (3) Change Research Methods
Smartphone Use		0.211 *** (0.060)	−0.499 *** (0.105)
Internet Use	−0.171 *** (0.037)		
Control Variables	Controlled	Controlled	Controlled
Regional Fixed Effect	Controlled	Controlled	Controlled
Sample Size	4351	4187	4351
Statistics	F = 38.45 ***	F = 31.72 ***	Wald = 7855.27 ***
R^2^	0.1128	0.1369	0.0603

Note: *** *p* < 0.01.

## Data Availability

All the original data can be obtained from the official website of CLASS, http://class.ruc.edu.cn (accessed on 15 April 2023). The identified analysis dataset is available to other researchers and others upon request by emailing the corresponding author (wxiaohui828@163.com).
